# Evaluating association between linguistic characteristics of abstracts and risk of bias: Case of Japanese randomized controlled trials

**DOI:** 10.1371/journal.pone.0173526

**Published:** 2017-03-09

**Authors:** Daisuke Yoneoka, Erika Ota

**Affiliations:** 1 Graduate school of Medicine, University of Tokyo, Tokyo, Japan; 2 St. Jude Children's Research Hospital, Department of Epidemiology and Cancer Control, Memphis, Tennessee, United States of America; 3 Graduate School of Nursing Science, St Juke’s International University, Tokyo, Japan; University of Ottawa, CANADA

## Abstract

Despite the ongoing growth in the number of published randomized controlled trials (RCTs) and increased quality assessment of RCTs, the association between the quality and characteristics in the text has not been sufficiently studied. We are interested in a specific question: what kind of sentences is a good indicator of high quality RCTs? To help researchers to efficiently screen articles worth reading, this study aims 1) to quantify the linguistic features of articles and 2) to build a document assessment model to evaluate quality of RCTs using only the abstract. All RCTs that were conducted in Japan in 2010 as original articles were included in the analysis. Data were independently assessed by two reviewers using a risk-of-bias tool. Three aspects of linguistic style were quantitatively measured, and a document model was constructed to evaluate the RCTs. A total of 302 RCTs were selected for quality assessment. Of these, 255 articles were assessed as high quality and 47 as low quality. High-quality articles tended to use longer words than low-quality articles (p = 0.048), however sentences were generally shorter (p = 0.004). Further, high-quality articles included a larger proportion of noun phrases (p = 0.026) but a smaller proportion of verb phrases (p = 0.041). The optimal number of topics to assess the quality of articles was four, while two topics had a significant association with quality. Despite a number of articles published about RCTs in Japan, significant differences exist in several textual features between high- and low-quality RCTs. Instead of the risk-of-bias tool, these results can be used as the new criteria to rapidly screen valuable articles and it also revealed quality control of RCT articles is urgently needed in Japan.

## 1. Background

Randomized controlled trials (RCTs) are widely regarded as the most reliable evidence in medical science [1]. A significant number of RCTs have been implemented worldwide [2], and in the context of systematic review or meta-analysis, the quality of RCT implementation can be evaluated by several assessment tools, all of which prove the validity of RCTs depending on their underlying methods (e.g., blinding, intention-to-treat, etc.) [3]. It has also been shown that RCTs with poor methodology may lead to a biased estimation of the true effects of the intervention [4–6].

To improve the quality of RCTs, various methodological approaches have been proposed. The CONSORT Statement, which was developed to provide a guideline for authors to improve reporting, is one example [4,7]. Although many approaches have been put forward to support researchers in their evaluation of trials (i.e., how to assess the quality of papers, how to interpret the results of RCTs), it is still difficult for researchers to stay abreast of the rapidly increasing volume of articles and methodologies. When evaluating RCT articles, many researchers base their initial assessments of an article’s quality and relevance by screening the abstract. Therefore, it is important to consider whether a method for identifying high-quality articles from specific textual features of the abstract could improve efficiency in researchers’ everyday work.

Current evidence on methods to effectively screen the quality of studies in this way remains limited. Some previous studies in the area of science journalism have investigated ways to forecast an article’s quality by analyzing the text corpora [8,9], where the ultimate purpose has been to automatically evaluate article quality without any human assistance [10]. However, the association between an article’s linguistic features and RCT quality is yet to be sufficiently explored. As a first step to obtaining this goal, the development of a document model for quantitative evaluation of RCTs using only abstracts for linguistic analysis offers a promising approach, because such a model could predict and recommend potentially high-quality articles for busy researchers to investigate further.

Therefore, the aims of this study are: 1) to analyze the association between various linguistic factors and the quality of RCTs, and 2) to build a document assessment model to evaluate quality of RCTs using only the abstract, which can help researchers who use only abstracts to identify potentially important articles more efficiently.

## 2. Methods

We searched both Japanese and non-Japanese databases for RCTs conducted in Japan and published in 2010 (S1 Table). Since this study analyzed the secondary data of our previous results, details of the search strategy and study selection were well documented in the previous study [11]. Yoneoka et al. (2014) examined the quality of Japanese RCT in 2010 using the risk of bias tool and provided detailed tables by the type of RCTs. To refer the results of previous classification of Japanese RCTs, we restricted this study to the year of 2010. We searched the domestic Japanese database, the Japan Medical Abstract Society Database, and the following international databases: MEDLINE, EMBASE, CINAHL and PsycINFO. Search terms related to RCTs such as ‘randomization’ and ‘double blind’ were incorporated into the search terms (final search: November 11, 2011). Search strategies were appropriately modified for each database in consultation with a librarian. All papers conducted in Japan that were published as original articles in 2010, with abstracts written in English, were included. Study protocols, conference abstracts and commentaries, and quasi-RCTs where allocation was conducted by date of birth, day of the week or medical record number, were excluded from this study. Lastly, characteristics data were extracted for each trial.

A Cochrane risk-of-bias tool was used to assess article quality. The assessed articles were randomly selected from all identified RCTs (i.e., 30% of articles were randomly selected). Developed by the Cochrane Back Pain Review Group, this risk-of-bias tool consists of 12 criteria for assessing the quality of an RCT with three items: “yes” (low risk of bias), “no” (high risk of bias) and “unsure” [12]. Studies were judged to be “low risk of bias” (i.e., high-quality articles) when at least six of the 12 criteria were scored as “yes”. Studies that scored “yes” for less than six criteria were rated as “high risk of bias” (i.e., low-quality articles). Two independent reviewers conducted this assessment. It is worth noting that while the quality assessment of the articles was conducted using both the abstract and the main text, the linguistic analysis was limited to the abstract data. This is because our study aimed to provide assessment criteria for rapid screening of articles for researchers who tend to only read the abstracts of articles.

We quantitatively measured three different aspects of linguistic style (baseline metrics, syntactic features and readability metrics, and lexical choice and diversity measures) [13] and constructed a document assessment model to simplify future evaluations of RCTs. Pearson’s correlation test was applied to assess the quality of the article (i.e., correlation with the number of “yes” items in the 12 criteria of the risk-of-bias tool) and Welch’s test was used to compare the means between high- and low-quality articles. A significant p-value was set at 0.05.

### 2.1 Baseline metrics

We tested the following metrics: 1) the average number of characters per word; 2) the average number of words per sentence; 3) the average number of sentences per 100 words, and 4) word count.

### 2.2 Syntactic features and readability metrics

The following metrics were assessed according to the degree of article quality: 1) average proportion of nouns (including numbers and names) per all words; 2) average proportion of verbs per all words, 3) average proportion of conjunctions per all words, and 4) Flesch Readability Score. The Flesch score formula is widely used and is regarded as the most reliable readability metric [14]. These four metrics are related to processing difficulty, and thus can affect the readability of the article.

### 2.3 Lexical choice based on n-gram and diversity measures

The N-gram (that means a sequence of n words) method was applied to assess lexical features; in particular, the unigram and bigram were analyzed [15,16]. The N-gram model was used to generate a list of most frequent sequence of n words in the collected articles. Further, as an indicator of lexical diversity, Herdan’s C index (sometimes referred to as LogTTR) was calculated [17]. Lexical diversity means the displayed range of vocabulary in a given text. High diversity indicates the greater range of different words in the text.

### 2.4 sLDA for evaluation of article quality

Supervised latent dirichlet allocation (sLDA) was used to model the documents in our corpus, which contained the selected abstracts [18]. LDA is a type of topic model and, in its unsupervised form, is a hierarchical Bayesian model for text analysis, which was firstly developed by Blei et al. (2003) [19]. Here, the term “topics” means clusters of words. The first stage of the hierarchical model represents each document that can be modeled as a finite mixture of latent sets of topics, assuming that a document with many words can be modeled as a composite of a small number of important topics. The second stage models each topic as a mixture of probabilities of the class of latent topic, which can define the probabilistic classification labels [20]. The sLDA model adds to this LDA model a response variable tagged to each document. This response variable is derived from the binarized result of risk of bias, taking 1 if the number of “yes” items in 12 risk-of-bias criteria exceeds 6; otherwise, it takes 0. In contrast to vanilla LDA, sLDA can simultaneously deal with pairs of documents and response variables or classification labels. In detail, the model allows the response variable and documents to be hierarchically modeled to represent the generative probabilistic structure, which can construct latent topics to efficiently predict response variables for future classification tasks (i.e., the future evaluation of the quality of RCTs). In this study, the last stage is formed as a logistic regression model with covariates of both topics and features connected to each document. The parameters (including number of topics) were tuned using a 10-fold cross-validation in terms of the maximize of the area under the curve (AUC). The following covariates were adjusted in the model: type of intervention (dummies indicating “device”, “biological/vaccine”, “procedure/surgery”, “physical therapy” and “drug and others”), disease and conditions based on ICD10 classification (dummies indicating “neoplasms”, “endocrine, nutritional and metabolic diseases”, “mental and behavioural disorders”, “diseases of the nervous system”, “diseases of the circulatory system”, “diseases of the respiratory system”, “diseases of the digestive system”, “diseases of the musculoskeletal system and connective tissue” and “others”), study design (dummies indicating “parallel”, “cross-over” and “factorial”), type of control group (dummies indicating “head to head”, “placebo” and “dose response”), number of arms (dummy variable takes 1 if number of arms ≥2, otherwise takes 0) and sample size. The model was build based on the stepwise model building technique. The discriminant performance of the models was measured by the area under the receiver operator characteristic curve (AUC) and the Brier score (BS), which are indicators of the accuracy of the model [21]. In addition, the model’s calibration was examined by the Hosmer-Lemeshow statistic choosing 10 groups [21].

## 3. Results

We found 2957 articles from Japan published in 2010 (EMBASE: 1174; Japan Medical Abstract database: 903; MEDLINE: 675; PsycINFO: 173 and CINAHL: 32). Of these, 1013 articles with English abstracts met our inclusion criteria and 1944 articles were excluded because of duplications (79.9%), non-RCTs (11.0%), and non-Japanese authorship (9.1%). Of the 1013 articles that met our inclusion criteria, 302 were randomly selected to proceed to the next step of quality assessment using risk-of-bias tools (S2 Table). From this number, 255 articles were judged as high quality and 47 were judged as low quality. Detailed results of classification are described in Yoneoka et al. (2014) [11].

Table 1 summarizes the results of the baseline measures. None of the baseline metrics were close to being significant predictors of article quality (i.e., the number of “yes” items in the risk-of-bias tool), however, the following comparisons between high- and low-quality articles showed significant results: the average number of characters per word (high: 5.46, low: 5.34, p = 0.048), the average number of words per sentence (high: 16.29, low: 19.16, p = 0.004), and the average number of sentences per 100 words (high: 6.52, low: 5.72, p = 0.003).

**Table 1:** Summaries of baseline measures.

	All articles*	Correlation	p-value **	Low quality	High quality	p-value ***
Average number of characters per words (words)	5.45	0.003	0.954	5.34	5.46	0.048
Average number of words per sentence (words)	16.73	-0.002	0.979	19.16	16.29	0.004
Number of sentences per 100 words (sentences)	6.40	-0.049	0.400	5.72	6.52	0.003
Number of characters per 100 words (characters)	544.53	0.003	0.954	534.04	546.46	0.048
Word count (words)	241.36	0.091	0.116	241.32	241.36	0.996

* Total articles: 302, (Low quality:47, High quality: 255).

** P-values for Pearson’s correlation test.

*** P-values for Welch’s test between low- and high-quality articles

Syntactic constructions are presented in Table 2. The average proportion of nouns and verbs showed significant results (high: 38.54%, low: 37.07%, p = 0.026 and high: 9.39%, low: 10.08%, p = 0.041, respectively).

**Table 2:** Summaries of syntactic features and readability score.

	All articles*	Correlation	p-value **	Low quality	High quality	p-value***
Average proportion of nouns (including numbers and names) per all words (%)	38.31	-0.017	0.768	37.07	38.54	0.026
Average proportion of verbs per all words (%)	9.50	0.055	0.340	10.08	9.39	0.041
Average proportion of conjunctions per all words (%)	3.59	-0.107	0.063	3.77	3.56	0.327
Flesch Readability Score	29.99	-0.017	0.764	30.91	29.82	0.569

* Total articles: 302, (Low quality:47, High quality: 255).

** P-values for Pearson’s correlation test.

*** P-values for Welch’s test between low- and high-quality articles

**Fig 1:**
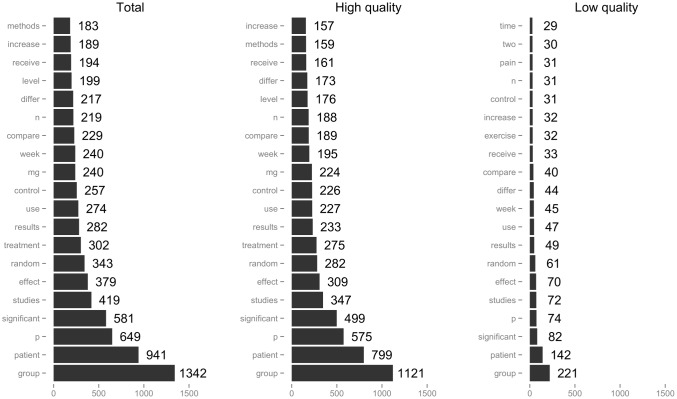
Top 20 results of unigram by article quality.

**Fig 2:**
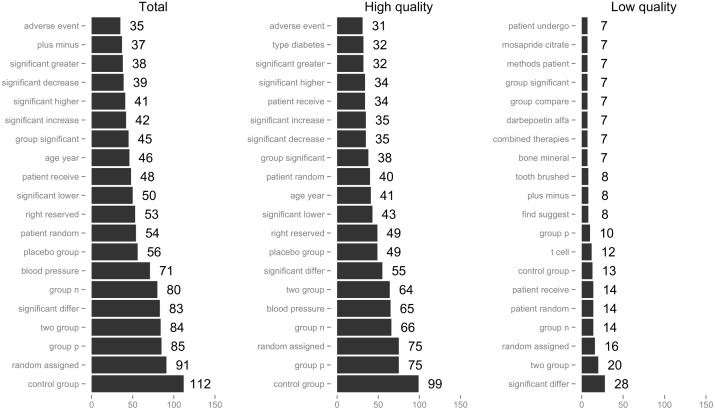
Top 20 results of bigram by article quality.

Figs 1 and 2 provide the top 20 most frequently reported words calculated from uni-/bi-gram, respectively. The top 5 most frequently used words (unigram) were similar both in high- and low-quality articles: “group”, “patient”, “significant”, “p” and “studies”. The top 5 most frequently used bigrams had relatively different sets in high- and low-quality articles: high (“control group”, “group p”, “random assigned”, “group n” and “blood pressure”); and low, (“significant differ”, “two group”, “random assigned”, “group n” and “patient random”), respectively. In terms of the Herdan’s C index for lexical diversity, the C index of total, high- and low-quality articles were 0.887, 0.888 and 0.885, respectively. Welch’s test between high- and low-quality articles did not show significant differences (p = 0.183), and Pearson’s correlation test also did not show a significant correlation (p = 0.764).

According to the results of the sLDA model, the optimal estimated number of topics was four, as follows: topic 1, “Outcome”; topic 2, “Surgery”; topic 3, “Procedure”, and topic 4 “Patient”. The optimal number of topics was selected based on the maximization of the AUC during the 10-fold cross-validation. Table 3 shows the top 10 frequently reported words for each of these four topics. The four topics and the covariates tagged in the articles were adjusted to determine the estimated coefficients from the sLDA model for article quality, shown in Table 4. Topic 2 “Surgery” and topic 3 “Procedure” had a significant association for article quality (adjusted odds ratio [AOR] was 3.44 [p = 0.049] for topic 2, and 7.35 [p = 0.013]) for topic 3, which means articles on these topics tend to increase the probability of being high-quality articles compared with articles on topic 1 “Outcome”. In terms of the prediction performance, the AUC was 76.95, the BS was 11.52, and the Hosmer-Lemeshow statistic was 4.62 (p = 0.797), which indicates no evidence of poor fitting of the model. In terms of positive and negative prediction values (PPV and NPV), the sLDA model provided 12.8% for PPV and 98.4% for NPV, respectively.

**Table 3:** Top 10 frequently reported words in detected topics of sLDA model.

Topic 1	Topic 2	Topic 3	Topic 4
"Outcome"	"Surgery"	"Procedure"	"Patient"
decrease	surgeries	placebo	mg
serum	postoperative	subject	patient
pressure	pain	exercise	cancer
blood	use	week	efficacies
level	propofol	trained	treatment
hypertensive	anesthesia	gastric	therapies
therapies	undergo	concentration	week
mm	infused	glucose	h
cardiovascular	record	weight	primarily
change	analgesia	bodily	oral

**Table 4:** Estimated coefficients of four topics from sLDA model.

	OR *	95% CI **	p-value
Topic 1	Ref.	-	-
Topic 2	3.44	(1.00, 11.79)	0.049
Topic 3	7.35	(1.53, 35.33)	0.013
Topic 4	1.19	(0.31, 4.58)	0.799

Adjusted covariates: type of intervention, disease and conditions, study design, type of control group, number of arms, sample size.

*OR: odds ratio

**CI: confidence interval

## 4. Discussion

This is the first study to evaluate the association between linguistic characteristics of abstracts and the quality of papers reporting RCTs conducted in Japan. Despite the considerable number of published articles from Japan, there is a large difference in textual features between high- and low-quality articles indicating that greater quality control of RCT articles is urgently needed in Japan.

One noticeable characteristic found in Japanese articles reporting on RCTs was that high-quality articles tended to use longer words than low-quality articles, however whole sentences in high-quality articles tended to be shorter. Since the usage of longer words within shorter sentences is widely perceived to indicate a well-written and informative article [13], authors writing about high-quality RCTs are likely to follow this principle to efficiently convey their results to the reader. Despite no differences in lexical diversity between high- and low-quality articles, the significantly larger proportion of noun phrases and smaller proportion of verb phrases in high-quality articles are somewhat surprising and interesting. The huge number of noun phrases forces readers to remember more items, but could make the abstract more engaging [13]. Further, while including multiple verb phrases helps readers to understand the structure of RCTs, it increases the sentence complexity in the abstract. Therefore, through the use of many noun phrases, high-quality articles are likely to engage the quick reader who reads only the abstract, and avoid complexity by using a small proportion of verbs. In addition, while surface metrics such as the average number of characters per word showed the potential as a predictor to distinguish high- or low-quality articles, they showed no highly significant association with the number of “yes” items in the risk-of-bias tools. Therefore, the results from the sLDA model, with several covariates adjusted, are worth considering. Topic 2 “Surgery” and topic 3 “Procedure” had a significant positive impact on quality, indicating a specific range of words that are preferred by authors of high-quality articles. However, it is necessary to reassess the role of these topics in other abstracts, which we plan to address in a future work. We welcome the re-evaluation of our model in other practical datasets. By using these linguistic features as the criteria to select the valuable articles, researchers can avoid to evaluate the quality of article with the risk-of-bias tool, which is sometimes a time consuming task for busy researchers.

Several limitations are present in our study. First, low quality of reporting was a critical obstacle in the assessment of article quality. As previously discussed [11], a large proportion of “unsure” results were observed in each risk-of-bias item, which hinders reviewers in evaluating the actual quality of RCTs. Therefore, it is necessary to construct a model that includes this uncertainty, such as a multi-labeled sLDA model, which we intend to explore in a future study. Second, the same weightings were applied to calculate the final quality of the abstract (i.e., simple average of the 12 criteria of the risk-of-bias tools). In this study, the quality of articles was binarized based on the number of “yes” items from the 12 criteria, which was calculated as a simple average of “yes” items. However, the importance of each criterion may differ according to the purpose of the reader. In such a case, we suggest a re-assessment of article quality based on the reader’s purpose using modified weights prior to linguistic interpretation. In addition, the definition “high quality article” as having a score of at least 6 items out of 12 items of the risk of bias tool should be re-considered: since the binarization of the quality score by a certain threshold may omit important information, the domain-based approach, which consider each domain of the risk of bias separately based on the reader’s preference, should be promoted. The third limitation is that our study examined only Japanese RCTs and was limited to the year 2010. However, considering relatively large contribution by Japanese to the evaluation of healthcare interventions, this attempt has a potential to be generalized to other influential countries [11]. We welcome the re-evaluation of our results in other journals or countries and in future we aim to detect trends in hot and cold topics by extending this methodology to a time-dependent structure.

Lastly, to improve the quality of RCT articles, the CONSORT statement should be more widely adopted by authors and publishers [7]. Several studies have shown that journal endorsement of CONSORT was positively associated with improvements in reporting RCTs [4], and pre-CONSORT trials have been found to be substandard in aspects of methodology compared to post-CONSORT studies [22,23]. Greater efforts are necessary to improve the reporting quality of RCTs, as well as further research of the linguistic aspects of medical articles [11]. Overcoming these difficulties will lead to better standardization of quality across abstracts, and enhance quality control.

## 5. Conclusions

This study revealed the current situation of the quality of Japanese RCTs, and found linguistic disparities between high- and low-quality articles based on Cochrane risk-of-bias tools. These linguistic results can be used as the criteria to screen potentially valuable articles and we recommend for researchers in medicine to apply these criteria to reduce the screening time for articles. To promote efficient screening of articles for busy researchers, further linguistic study and quality control of RCT articles are urgently needed.

## Supporting information

S1 Table PRISMA check list. (DOC)

S2 Table Main dataset of selected articles. (CSV)

## References

[1] Higgins J.P. & Green S. Cochrane handbook for systematic reviews of interventions: Wiley Online Library, 2008.

[2] Gluud C. & Nikolova D. Likely country of origin in publications on randomised controlled trials and controlled clinical trials during the last 60 years. Trials, 2007, 8, 7 10.1186/1745-6215-8-7 17326823 PMC1808475

[3] Strippoli G.F., Craig J.C. & Schena F.P. The number, quality, and coverage of randomized controlled trials in nephrology. Journal of the American Society of Nephrology, 2004, 15(2), 411–9. 14747388 10.1097/01.asn.0000100125.21491.46

[4] Moher D., Cook D.J., Jadad A.R., Tugwell P., Moher M. & Jones A. Assessing the quality of reports of randomised trials: implications for the conduct of meta-analyses. Health Technol Assess, 1999, 3(12), i–iv, 1–98. 10374081

[5] Juni P., Altman D.G. & Egger M. Systematic reviews in health care: Assessing the quality of controlled clinical trials. BMJ, 2001, 323(7303), 42–6. 11440947 10.1136/bmj.323.7303.42 PMC1120670

[6] Wood L., Egger M., Gluud L.L., Schulz K.F., Juni P. & Altman D.G. Empirical evidence of bias in treatment effect estimates in controlled trials with different interventions and outcomes: meta-epidemiological study. BMJ, 2008, 336(7644), 601–5. 10.1136/bmj.39465.451748.AD 18316340 PMC2267990

[7] Schulz K.F., Altman D.G., Moher D. & Group C. CONSORT 2010 statement: updated guidelines for reporting parallel group randomised trials. Int J Surg, 2011, 9(8), 672–7. 10.1016/j.ijsu.2011.09.004 22019563

[8] Druck G., Miklau G. & McCallum A. Learning to predict the quality of contributions to wikipedia. WikiAI, 2008, 8, 7–12.

[9] Louis A. & Nenkova A. A corpus of science journalism for analyzing writing quality. Dialogue and Discourse, 2013, 4(2), 87–117.

[10] Eysenbach G. & Diepgen T.L. Towards quality management of medical information on the internet: evaluation, labelling, and filtering of information Hallmarks for quality of information Quality on the internet Assuring quality and relevance of internet information in the real world. BMJ, 1998, 317(7171), 1496–502. 9831581 10.1136/bmj.317.7171.1496 PMC1114339

[11] Yoneoka D., Hisashige A., Ota E., Miyamoto K., Nomura S. & Segawa M. Are Japanese randomized controlled trials up to the task? A systematic review. PLoS One, 2014, 9(3), e90127 10.1371/journal.pone.0090127 24595104 PMC3940821

[12] Furlan A.D., Pennick V., Bombardier C., van Tulder M. & Editorial Board C.B.R.G. 2009 updated method guidelines for systematic reviews in the Cochrane Back Review Group. Spine (Phila Pa 1976), 2009, 34(18), 1929–41. 19680101 10.1097/BRS.0b013e3181b1c99f

[13] Pitler E. & Nenkova A. Revisiting readability: A unified framework for predicting text quality. Proceedings of the Conference on Empirical Methods in Natural Language Processing: Association for Computational Linguistics; 2008. p. 186–95.

[14] DuBay W.H. The Principles of Readability. Online Submission; 2004.

[15] Louis A. & Nenkova A. What makes writing great? First experiments on article quality prediction in the science journalism domain. Transactions of the Association for Computational Linguistics, 2013, 1, 341–52.

[16] Ashok V.G., Feng S. & Choi Y. Success with style: Using writing style to predict the success of novels. Poetry, 2013, 580(9), 70.

[17] Herdan G. Linguistic philosophy in the light of modern linguistics. Language and Speech, 1960, 3(2), 78–83.

[18] Mcauliffe J.D. & Blei D.M. Supervised topic models. Advances in neural information processing systems.; 2008 p. 121–28.

[19] Blei D.M., Ng A.Y. & Jordan M.I. Latent dirichlet allocation. the Journal of machine Learning research, 2003, 3, 993–1022.

[20] Cardenas A.F., Buttler D.J. & Critchlow T.J. Measuring the interestingness of articles in a limited user environment. Information Processing & Management, 2011, 47(1), 97–116.

[21] Pepe M.S. The statistical evaluation of medical tests for classification and prediction. [pbk. ed. Oxford; New York: Oxford University Press, 2004.

[22] Plint A.C., Moher D., Morrison A., Schulz K., Altman D.G. & Hill G. Does the CONSORT checklist improve the quality of reports of randomised controlled trials? A systematic review. Medical Journal of Australia, 2006, 185(5), 263–67. 16948622 10.5694/j.1326-5377.2006.tb00557.x

[23] Turner L., Shamseer L., Altman D.G., Weeks L., Peter J. & Kober T. Consolidated standards of reporting trials (CONSORT) and the completeness of reporting of randomised controlled trials (RCTs) published in medical journals. Cochrane Database Syst Rev, 2012, 11, MR000030 10.1002/14651858.MR000030.pub2 23152285 PMC7386818

